# Ecoepidemiological and Social Factors Related to Rabies Incidence in Venezuela during 2002-2004

**Published:** 2006-02

**Authors:** Pedro M. Rifakis, Jesus A. Benitez, Alfonso J. Rodriguez-Morales, Sonia M. Dickson, Jose De-La-Paz-Pineda

**Affiliations:** 1 *Division of Internal Medicine and Epidemiology, Hospital Perez de Leon, Health Direction of Municipio Sucre, Miranda, Caracas, Venezuela;*; 2 *Enviromental Health Office, Ministry of Health and Social Development, Maracay, Aragua, Venezuela;*; 3 *Instituto Experimental José Witremundo Torrealba, Universidad de Los Andes, Trujillo, Venezuela*

**Keywords:** Rabies virus, ecoepidemiology, climate variability, social factors

## Abstract

Rabies in Venezuela has been important in last years, affecting dogs, cats, and human, among other animals, being a reportable disease. In Zulia state, it is considered a major public health concern. Recently, a considerable increase in the incidence of rabies has been occurring, involving many epidemiological but also ecoepidemiological and social factors. These factors are analyzed in this report. During 2002-2004, 416 rabies cases were recorded. Incidence has been increasingly significantly, affecting mainly dogs (88.94%). Given this epidemiology we associated ecoepidemiological and social factors with rabies incidence in the most affected state, Zulia. In this period 411 rabies cases were recorded. Zulia has varied environmental conditions. It is composed mostly of lowlands bordered in the west by mountain system and in the south by the Andes. The mean is temperature 27.8°C, and mean yearly rainfall is 750 mm. Climatologically, 2002 corresponded with El Niño (drought), middle 2003 evolved to a Neutral period, and 2004 corresponded to La Niña (rainy); this change may have affected many diseases, including rabies. Ecological analysis showed that most cases occurred in lowland area of the state and during rainy season (*p*<0.05). Additionally, there is an important social problem due to educational deficiencies in the native population. Many ethnic groups live un Zulia, many myths about rabies are in circulation, and the importance of the disease is not widely realized. The full scale of the rabies burden is unknown, owing to inadequate disease surveillance. Although there have been important advances in our knowledge and ability to diagnose and prevent it, enormous challenges remain in animal rabies control and provision of accessible-appropriate human prophylaxis worldwide. Human and animal surveillance including ecological and social factors is needed.

## INTRODUCTION

Lyssaviruses, such as that which causes rabies, are negative strand RNA viruses that can be divided into seven genotypes (1-3). Viruses of genotypes 1, 5 and 6 are characterized by their natural and stable association with specific mammalian species, which act as vectors for their transmission, so that a number of phylogenetic lineages co-circulate among a range of mammalian species (1, 4). Infection of an animal with a lyssavirus that originated within a different reservoir population will generally lead to a fatal self-limiting rabies-like infection (a “spill-over”), as in the case of humans, and only occasionally to a new stable enzootic infection (1).

Additionally, other epidemiological factors should be considered in countries where rabies is a public health threat. Recently the ecoepidemiological and social factors have been considered in Venezuela for rabies as well for other endemic diseases such as malaria, dengue, and equine encephalitis, among others.

The burden of rabies in Venezuela has been important in the last 20 years. The disease affects dogs, cats, and human, other animals (e.g. equine and bovine). The main endemic zone of this disease has been a western state of Zulia (which borders Colombia), where is considered it a major public health concern (5-9). Recently, approaches such as ecoepidemiology ard landscape epidemiology are being considered in the assessment of disease situation and public health management.

The usefulness of landscape in this disease has was first described more than 20 years ago, when it was suggested that physiographic and ecological features may serve as barriers or as passageways for the epidemic spread of rabies (10). Epidemic spread is characterized by radial and linear patterns reflecting physiographic features. These patterns could be related to the fact that occasionally lyssaviruses gain access to new populations of susceptible hosts, particularly those which are geographically restricted (11, 12), or evolve to infect previously less susceptible hosts (13-16). It is evident that such an adaptive process took place in many places in the world, including Americas and Venezuela during the first decades of past century when rabies virus became established in the fox following a decline in incidence among urban dogs and wolves (1). The incidence of rabies in the different animals varies according to seasons. Present control efforts are generally ineffective; under certain conditions, animal population reduction for control of rabies may be detrimental instead of beneficial (10).

Given this scenario, new analyses could be useful in the management of disease and its control in an integral form. As has been stated, recently, a considerable increase in the incidence of rabies has been occurring in Venezuela, particularly in the western state of Zulia. In addition to epidemiological factors, ecological, ecoepidemiological, and social factors play roles in its spread. This report uses a landscape epidemiology approach to analyze the spread and distribution of rabies in Zulia.

## MATERIALS AND METHODS

Epidemiological data for this study were retrieved from records of the Ministry of Health from Venezuela. All these cases are clinically, epidemiologically, histopathologically and virologically studied and confirmed before report them to records. Geographical and climatological data were obtained from meteorological field stations and satellital geographical information systems (NOAA). The inter-annual variability of rabies incidence was analyzed. In this study we tried to identify ecoepidemiological and social factors related to rabies incidence in Venezuela during 2002-2004. We focus also in the differences of rabies incidence according to climate variability and El Niño Southern Oscillation (ENSO) events in those years.

**Figure 1:**
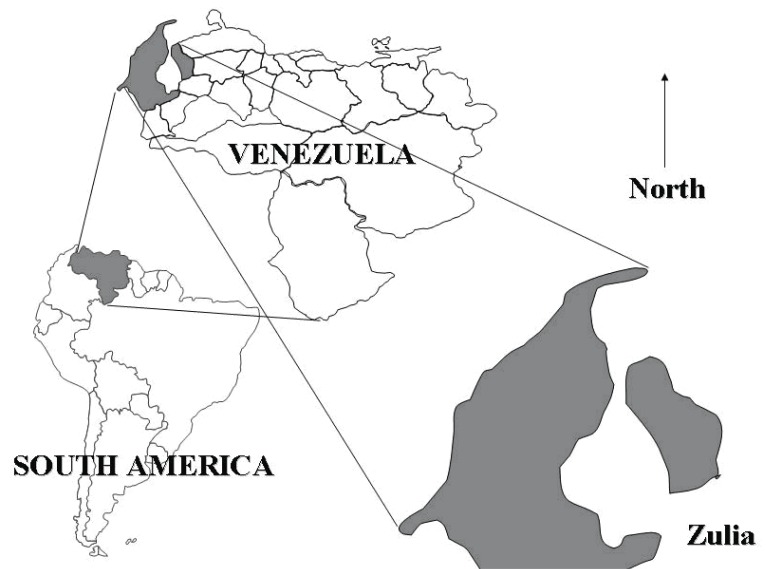
Map of Venezuela with relative position of Zulia (gray area).

Given this epidemiology we associated ecoepidemiological and social factors such as: climate variability, geographical conditions, socioeconomical issues (these were retrieved from the National Institute of Statistics, INE), among others, with rabies incidence in the most affected state, Zulia. The area of study is Zulia, which is a border state with Colombia in Western Venezuela (8°21’-11°51’N, 70°40’-73°25’W) (Fig. 1). The statistical analyses were performed with Epi Info v.6.0 with a level of confidence of 95%.

## RESULTS

**Figure 2:**
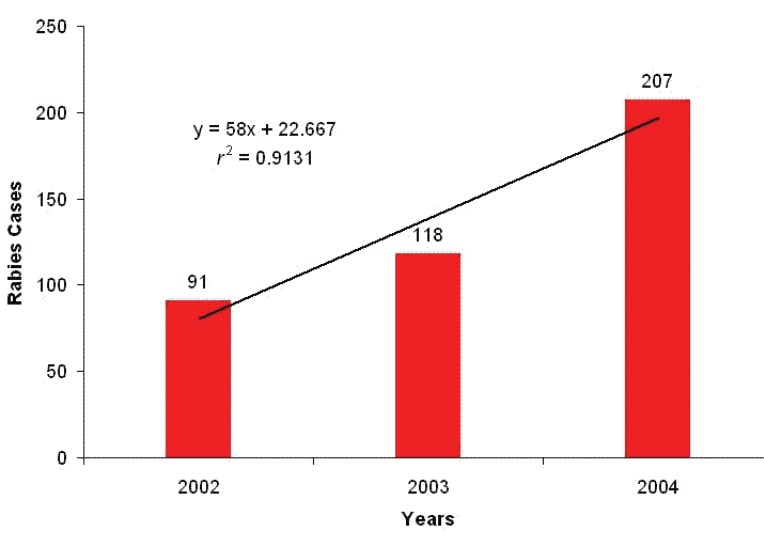
Rabies incidence in Venezuela during the period 2002-2004.

**Figure 3:**
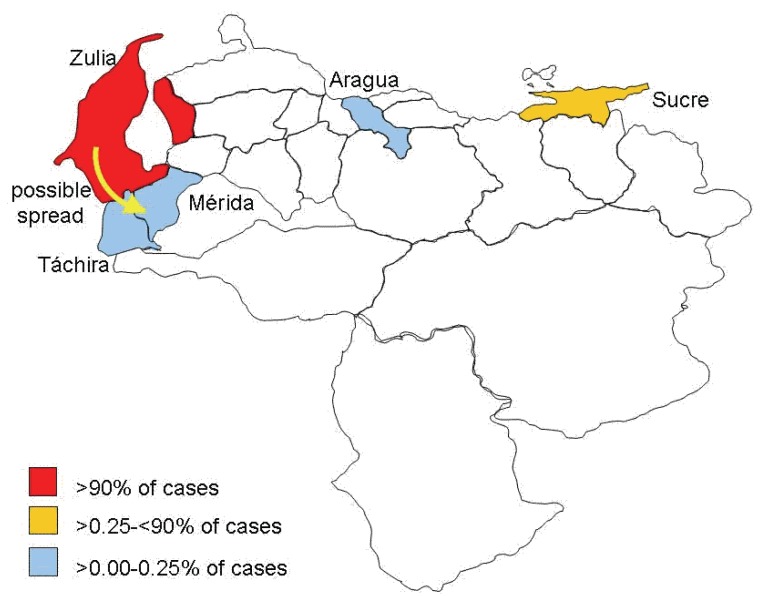
Rabies incidence distribution in Venezuela during the period 2002-2004.

**Figure 4:**
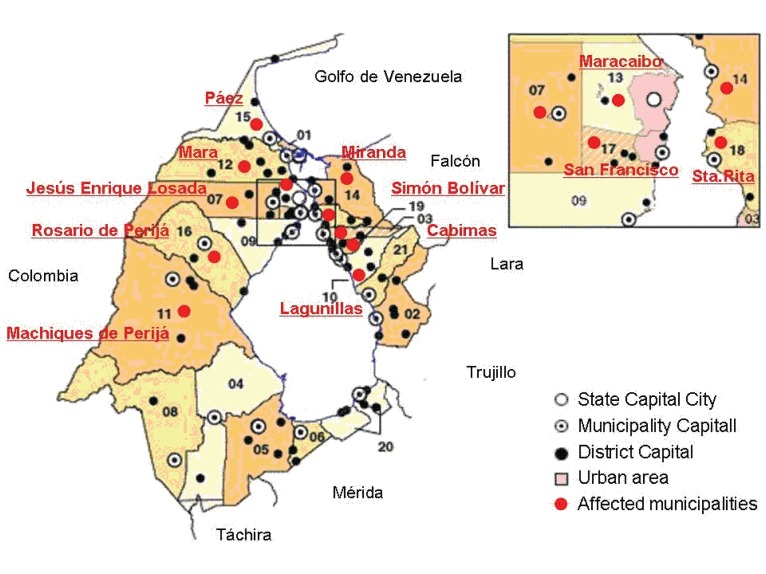
Rabies incidence distribution in Zulia, Venezuela during the period 2002-2004, showing the affected municipalities (insert: metropolitan area).

During 2002-2004, 416 rabies cases were recorded. Incidence has been considerably increasing, 2002: 91, 2003: 118, 2004: 207 (r^2^=0.913, *p*=0.191) (Fig. 2), with following state geographical distribution: 98.80% in Zulia, 0.48% in Sucre, 0.24% Táchira, 0.24% Mérida and 0.24% Aragua (χ^2^=2017.98; *p*<0.01) (Fig. 3); affecting mainly dogs (88.94% cases), cats (4.81%), humans (1.68%) and other animals (4.57%) (χ^2^=1210.85; *p*<0.01). As first cases of the period occurred in Zulia, it has been proposed the spread of disease to Táchira and Mérida was from that state by the southern are of the state (Fig. 3), although southern municipalities were not affected by rabies during this period (Fig. 4). Distant migration of human and animals could explain the occurrence of cases in Aragua and Sucre (Fig. 3).

In the studied period 411 rabies cases were recorded in Zulia (2.99 ± 2.44 [±SD] cases/week), affecting mainly dogs 89.5%, cats 4.6%, humans 1.2% (χ^2^=949.11; *p*<0.01). Zulia is a border state with Colombia in Western Venezuela (Fig. 1), it has varied environmental conditions, with 63,100 km^2^ (6.9% Venezuela surface), 3,209,000 habitants and 21 municipalities located around the Maracaibo lake (Fig. 4); they are communicate by a bridge on the lake between municipalities of San Francisco (municipality number 17, insert, Fig. 4) and (municipality number 18, insert, Fig. 4). From these municipalities, in 12 were recorded rabies cases, being most affected Cabimas (municipality number 03, Fig. 4) and Maracaibo (municipality number 13, Fig. 4) (>65% cases).

**Figure 5:**
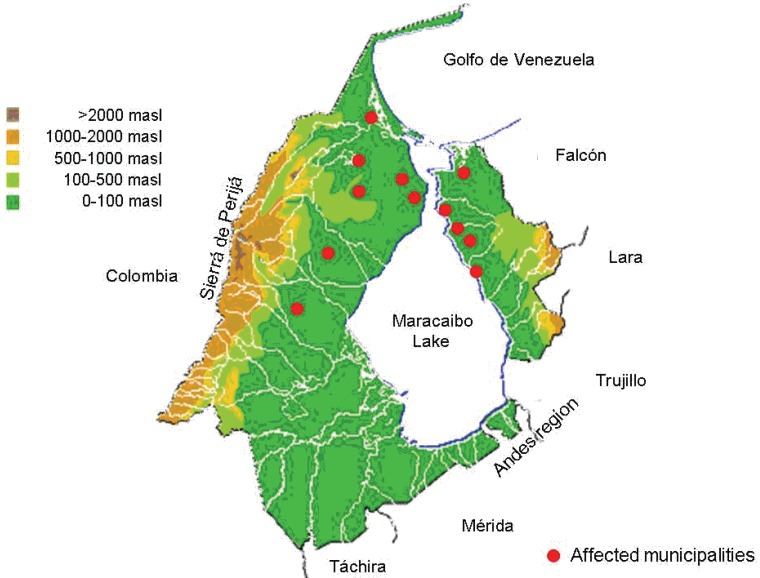
Physiographical and hydrographical map of Zulia showing the rabies incidence distribution in Zulia, Venezuela during the period 2002-2004, and the affected municipalities.

In regard to ecoepidemiological conditions of this region, Zulia state surface is mostly lowlands with a west border constituted by a mountain system (Sierra de Perijá, 3750 masl) and south border by the mountain system of Venezuelan Andes (Fig. 5). Land territory surrounds one of greater lakes (Maracaibo lake, 20.4% of state surface) (Fig. 5). Most rabies affected zones corresponded to municipalities with altitude <100 masl. Mean temperature of Zulia is 27.8°C and mean rainfall, 750mm (Fig. 4 and 5). Analyzing the macro-climatic conditions, the year 2002 corresponded with El Niño events (drought), but middle 2003 evolved to a Neutral period and finally 2004 to La Niña period (rainy); this change could affected many diseases, including rabies, due to changes in living conditions, for animals and humans. Probably these favorable conditions contribute with the increase of rabies incidence during the period 2002-2004.

Additionally, there is an important social problem due to education lack in this region (the rate of illiteracy is 8.2% of state population and for 2002 presented inside the State Life Level Index [0.7852], the lowest educational component [0.5547] for the country, considered at the current classification as a value of middle low level [0.5-0.59]). Many people live in rural areas and have access to neither education nor medical facilities (although the state reported of the higher human development index for 2001, 0.7519, but this is probably due to people living in Maracaibo, the capital city of the state, where life conditions differ significantly from the rest of the state of Zulia). This factor could be also contributing with the epidemiology of rabies in Zulia, because it is logistically impossible for people who live away from medical centers to receive vaccines on time. From the total number of families considered as poor for 2003 in Venezuela (2,985,332), most corresponded to Zulia (393,007) being the first state of the country in poorest families (13.16% of the country) (58.6% of families are poor). In Zulia live many ethnic groups (which reach 196.911 individuals for 1992, 62% of national indigenous population) (most important is the “Guajiros”), they believe in myths about rabies and not realize disease importance. All these ecoepidemiological and social factors should be now considered for the further study of disease in the region and other endemic zones in the country.

## DISCUSSIONS

Full scale of rabies global burden is unknown, owing to inadequate surveillance of disease, social and ecological factors as well for reservoirs. In the Americas, the bat species Desmodus rotundus (vampire bat), Tadarida brasiliensis (Brazilian free-tailed bat), Eptesicus fuscus (big brown bat), Lasiurus species (L. borealiz, L. cinereus), Lasionycteris noctivagans (silver-haired bat), Pipistrellus subflavus (eastern pipistrelle) and Myotis species (M. lucifugus; M. yumanensis, M. californicus; M. evotis) have all been identified as rabies virus (RV) reservoirs that harbour distinct RV variants (17-19). The role of bat species in the spread of rabies in Venezuela has not been recently studied (8), as well other factors such as social and ecological ones reported in this study for other endemic areas in the country.

The RV variants generally separate into phylogenetic divisions that represent the lifestyle of their chiropteran hosts, i.e. migratory versus non-migratory, colonial versus solitary, insectivorous versus haematophagus (17, 19). While most of these variants co-segregate only with their specific host reservoirs making spillover events to terrestrial animals uncommon, some have been associated with infection of non-chiropteran species, especially humans and domestic animals (17, 19-22). These genetic and molecular aspects also need to be addressed in Venezuela.

Although there have been important advances in our knowledge and ability to diagnose and prevent rabies (23, 24), enormous challenges remain in animal rabies control and provision of accessible-appropriate human prophylaxis worldwide (25); for Venezuela, as we seen in Zulia state, living conditions, poverty, education, the habits of indigenous cultures, access to health services, and also the climate variability and ecological factors, are playing a potential role in the contribution of rabies burden and incidence and persistence of disease in the region and the country.

There have been recent important advances in our understanding of how rabies virus spreads and causes disease in its hosts, but new insights are also required. More research is needed in good experimental animal models in order for us to better understand the pathogenesis of this ancient disease (26). Because current approaches to the management of human rabies have proven unsatisfactory (27), this knowledge may be important for the development of novel therapies for the treatment of rabies and other viral diseases in the future, which jointly with landscape epidemiology will give us an integral vision and tool for the management of this public health threat.

More human and animal surveillance including ecological and social factors is needed, and further research is expected.
